# The Connecticut Valley Dental Association

**Published:** 1866-12

**Authors:** 


### Proceedings of Societies.

### THE CONNECTICUT VALLEY DENTAL ASSOCIATION.

The Connecticut Valley Dental Association began its fourth annual meeting at Hayne's Hotel, Springfield, Mass., on Tuesday the 15th of October, with a good attendance of dentists from Vermont, western and eastern Massachusetts and Connecticut. The following members of the association were elected officers for the ensuing year: President, Dr. L. D. Shepard of Salem; vice presidents, C. A. Howland of Barre, and H. F. Bishop of Worcester; secretary, W. H. Jones, of Northampton; treasurer, A. F. Davenport of North Adams; executive committee, O. F. Harris, of Worcester, T. W. Meekins of Northampton and A. B. Cowan of Palmer. The retiring president, Dr. J. Beals of Greenfield, made an excellent address, reviewing the progress of the profession during his long practice, and embracing many facts both interesting and of eminent importance to members of the profession. The remainder of the afternoon session was occupied by a discussion of the "comparative value of soft foil and adhesive gold in durability of results.' This discussion was opened by Dr. I. J. Wetherbee, of Boston, who is quite eminent in the profession.

The evening session was well attended, and the afternoon subject was continued, occupying all the time until adjournment. The various members of the association related their experiences in the use and comparative value of soft foil and adhesive gold in durability of results, and formed a kind of class meeting which was freer than a parliamentary discussion, and much more conducive to draw out information on all sides. Late in the evening the meeting broke up, and the members were agreeably surprised and entertained by a bountiful collation provided by the proprietors of the hotel.

SECOND DAY.

A larger number of dentists than on the first day assembled at the morning session of the Dental Association on Wednesday, and the exercises throughout the day were both varied and interesting. The first exercise of the forenoon was a clinic before the association, three dentists, Dr. I. A. Salmon, of Boston, Dr. J. N. Davenport, of Northampton, and A. W. Davenport, of North Adams, officiating at filling the teeth of as many patients. The first topic under discussion was "Nitrous oxide, and other anæsthetics," and it was opened by an essay read by Dr. George Bowers, of Springfield, Vt., and participated in by nearly all present, in an off-hand, pleasant talk. The following resolution was then passed by unanimous vote:

"*Resolved*, That this association do not consider the nitrous oxide made by Sprague's arrangement any purer than may be produced by the apparatus not interfering with his patent, which applies only to the convenience of the operator in regulating the flame."

Dr. Salmon, of Boston, then read an essay on "Conservative Dentistry, or the best means for accomplishing our great aim, preserving the greatest number of natural teeth."

In the afternoon a code of ethics came up for consideration, being in substance the same as that adopted by the American Dental Association at their last meeting, and specifying the duties which dentists owe to each other, their patients, and the profession. Alveolar abscess was then discussed ably and fully by many of the members. It may not be known to the public generally that with the present advanced state of dental science, not only decayed, but even ulcerated teeth can be restored to health and usefulness, and this important fact was elicited in the discussion. The following essayists were appointed for the next session: Dr. A. A. Holland, of Barre, Dr. H. Wheeler, of Holyoke, Dr. T. W. Meekins, of Northampton, and Dr. Jairus S. Hurlburt of Springfield. An improved head-rest was exhibited to the association by Dr. O. C. White, of Hopkinton, the inventor, and was generally approved.

Resolutions, complimentary to the proprietor of Hayne's Hotel, and thanking him and his assistants for the many accommodations and favors received, were unanimously passed. The meeting of the association ended at dark, and it was voted that the next session be held at Northampton, on the second Tuesday in June, 1867.

